# My Health, My Life, My Way—An Inclusive Web-Based Self-management Program for People With Disabilities Living With Chronic Conditions: Protocol for a Multiphase Optimization Strategy Study

**DOI:** 10.2196/31694

**Published:** 2023-04-28

**Authors:** Eric Evans, Ayse Zengul, Amy Knight, Amanda Willig, Andrea Cherrington, Tapan Mehta, Mohanraj Thirumalai

**Affiliations:** 1 Department of Health Services Administration School of Health Professions University of Alabama at Birmingham Birmingham, AL United States; 2 Department of Nutrition Sciences School of Health Professions University of Alabama at Birmingham Birmingham, AL United States; 3 Department of Neurology Heersink School of Medicine University of Alabama at Birmingham Birmingham, AL United States; 4 Division of Infectious Disease Heersink School of Medicine University of Alabama at Birmingham Birmingham, AL United States; 5 Division of Preventive Medicine Heersink School of Medicine University of Alabama at Birmingham Birmingham, AL United States

**Keywords:** telehealth, health coaching, artificial intelligence, chronic conditions, mobile phone

## Abstract

**Background:**

Individuals with disabilities living with chronic health conditions require self-management programs that are accessible, sustainable, inclusive, and adaptable. Health coaching is an effective approach to promoting behavior change in self-management. Health coaching combined with telehealth technology has the potential to improve the overall quality of, and access to, health services.

**Objective:**

This protocol outlines the study design for implementing the My Health, My Life, My Way intervention. The study will assess the feasibility, acceptability, and preliminary efficacy of the intervention for people with disabilities and optimize it.

**Methods:**

The My Health, My Life, My Way study is a 4-arm randomized controlled trial evaluating the delivery of a 6-month intervention involving telecoaching, inclusive educational content, and technology access for 200 individuals with chronic conditions and physical disabilities. This study uses the engineering-inspired multiphase optimization strategy (MOST) framework to evaluate intervention components and assess whether a combination or lack of individual elements influences behavior. Participants will be randomized to 1 of 4 study arms: scheduled coaching calls and gamified rewards, no scheduled coaching calls and gamified rewards, scheduled coaching calls and flat rewards, and no scheduled coaching calls and flat rewards.

**Results:**

The My Health, My Life, My Way study was approved by the institutional review board of the University of Alabama at Birmingham, and recruitment and enrollment will begin in May 2023. Data analysis is expected to be completed within 6 months of ending data collection. This clinical trial protocol was developed based on the SPIRIT (Standard Protocol Items: Recommendations for Interventional Trials) 2013 statement.

**Conclusions:**

The My Health, My Life, My Way study will help to optimize and improve our understanding of the feasibility and efficacy of a web-based self-management program for people with physical disabilities and chronic conditions. More specifically, My Health, My Life, My Way will determine which combination of interventions (coaching calls and gamification) will result in increased participation in self-management programming. The My Health, My Life, My Way intervention has the potential to become a scalable and novel method to successfully manage chronic conditions in people with disabilities.

**Trial Registration:**

ClinicalTrials.gov NCT05481593; https://clinicaltrials.gov/ct2/show/NCT05481593

**International Registered Report Identifier (IRRID):**

PRR1-10.2196/31694

## Introduction

### Background

The Centers for Disease Control and Prevention (CDC) defines chronic diseases as conditions that last ≥1 year and require ongoing medical attention or limit activities of daily living or both. In the United States, 6 in 10 adults are reported to have at least 1 chronic condition, whereas 4 in 10 adults are reported to have ≥2 chronic conditions [1]. The prevalence statistics of these individual conditions, which include diabetes, heart disease, and stroke, present an even more disturbing picture; for example, diabetes affects 12.2% of the overall American adult population and 25.25% of the population aged >65 years [1,2]. Similarly, approximately 1 in 3 deaths in the United States are attributed to heart disease, stroke, and other cardiovascular diseases [3]. In 2020, the CDC identified chronic diseases as the leading causes of death and disability as well as the leading drivers of annual health care costs of US $3.5 trillion [1].

Although physical disabilities increase the risk of developing chronic conditions, the inverse also occurs [4,5]. In fact, chronic conditions are the leading causes of disability [1]; for example, a report on the top causes of disability among adults in the United States found arthritis, heart conditions, and diabetes among the top 6 causes of disability [6]. This bidirectional relationship between disabilities and chronic conditions is indeed well documented. Diabetes is associated with a substantial increase in the risk of mobility disability [4]. Among older participants with diabetes (aged ≥60 years), diabetes was correlated with a 2- to 3-fold increased odds of not being able to perform each task of walking one-fourth of a mile, climbing stairs, or doing housework among both men and women [2]. In brief, chronic conditions and disability exist in a tightly knit closed loop, with the onset of any of these factors exposing the individual to increased risk for the other.

Current science has enabled multiple approaches to managing chronic disease conditions [7,8]. Approximately two-thirds of chronic disease management is conducted in primary care settings globally, whereas approximately one-fifth is conducted in community-based settings [9]. An extensive review of 145 chronic disease management studies notes that self-management support is the most frequently used intervention approach and the most effective strategy [7]. A meta-analysis specifically focused on chronic disease self-management strategies for diabetes, hypertension, and osteoarthritis spanning 53 randomized controlled trials concludes that these programs are indeed effective for people with diabetes or hypertension [10].

Within these programs, multiple approaches have been used to implement self-management programs. Health coaching is one of the most common and cost-effective mechanisms of delivering self-management programming [11-13]. Health coaches are health care professionals who are trained in relevant areas of focus based on the disease or condition being managed. Positive aspects of health coaching include removal of barriers and increased retention with human interactions that support individualized interventions. Health coaching can be conducted either synchronously or asynchronously, which further allows individualization to meet participant needs. However, health coaching has 2 challenges—resources and availability of time spent with participants—both of which affect the scalability of health coaching [14]. In addition to health coaching, gaming features incorporated into telehealth interventions have the capability to promote adherence and compliance and warrant adoption in disability-related self-management programs. Such features include obtaining points, participating in challenges or competitions, social communication, and person-centered customizations such as avatars. There is broad appeal to incorporating gaming elements into self-management programs for individuals with physical disabilities [15].

Although self-management programs are effective for behavior change in managing health conditions, individuals with physical disabilities who also experience chronic conditions lack inclusive and accessible self-management programming to positively affect their own behavior change. People with disabilities experience barriers that are different from those experienced by people without physical disabilities, including nutritional and dietary concerns, as well as barriers to physical activity (PA) participation. More specifically, these barriers include lack of accessible environments, appropriate equipment (eg, wheelchair accessibility), and low health literacy and knowledge [16-19]. As there are few studies evaluating the impact of self-management programs on individuals with physical disabilities, there is a demand to provide and optimize holistic self-management programming to those with physical disabilities who also experience chronic conditions.

In the goal to improve chronic disease management for people with disabilities, we propose the development of an inclusive telecoaching self-management program. To make the telecoaching approach sustainable and scalable, artificial intelligence techniques are used to reduce the time that health coaches spend communicating with each participant.

### Objectives

This study is structured to serve as the optimization phase of the multiphase optimization strategy (MOST) framework and involves efficient randomized experimentation to estimate the effectiveness of individual intervention components and identify the optimal intervention package to improve the primary outcome, health-related quality of life after 6 months [20,21]. We will specifically focus on the physical functioning and emotional well-being subscale scores. The feasibility and acceptability of the My Health, My Life, My Way intervention will also be studied. This study protocol has been developed by using the SPIRIT (Standard Protocol Items: Recommendations for Interventional Trials) 2013 statement [22]. It has been registered at ClinicalTrials.gov (NCT05481593).

## Methods

### Study Overview

#### Coaching Calls

Telehealth programs provide several advantages for individuals who want to improve their overall health and promote positive behavior change [23]. A substantial advantage of such programs is scalability or widespread availability, which allows participants to engage domestically or internationally. Many of these programs use human-to-human interactions to deliver web-based content to their clients or patients. In addition, some programs now implement artificial intelligence in conjunction with human interactions; for example, certain health care industries use chatbots to provide satisfactory and engaging interactions. A chatbot is a software platform that mimics human interaction with individuals using text or voice capabilities across different technologies, such as computers and smartphones. Within the literature, there is an acceptable level of satisfaction with using chatbot technology [24,25]. Because of the perceived benefits of telehealth programs using human interactions as well as web-based chatbots, it is necessary to evaluate whether scheduling coaching calls are necessary for successful self-management of chronic conditions. Each study arm in this project will incorporate either scheduled coaching calls or no scheduled coaching calls. Those receiving scheduled coaching calls will receive weekly calls with trained health coaches. Although individuals not receiving coaching calls will not have regular contact with a health coach, these participants can still contact health coaches using texting, chatting, or calling to initiate on-demand communication with the health coaching team.

#### Gamification

Gaming technologies have displayed a high level of adherence and engagement characteristics, and this has led to the gamification of telehealth interventions. Gamification refers to the use of game design elements in nongame contexts and is being used across a variety of telehealth interventions to promote treatment adherence [15]. Within telehealth intervention, gamification elements provide immediate feedback, continuous progress reporting, points, badges, levels, challenges, competitive components, and avatar and profile functionality [15]. There is a broad appeal to including gamification elements in telehealth studies for multiple reasons, such as (1) supporting transition to increased levels of intrinsic motivation, (2) deliverability through multiple technology streams, (3) appeal across different audiences, and (4) appeal in health and fitness industries [15]. However, the impact of gamification features on redeemable rewards is unclear [26]. More specifically, it is unknown how individuals’ intrinsic motivation will be affected by redeemable rewards. Within this study, participants will receive either gamified rewards or flat rewards. For those receiving gamified rewards, there will be an increase in the number of redeemable points the longer adherence and participation are maintained. Those receiving flat rewards will receive a fixed number of points for regular participation in the intervention. Elements of participation include but are not limited to logging in to the platform, interacting with the educational content, and watching multimedia content. These variations in rewards will inform whether gamification features alone promote a shift from extrinsic to more intrinsic motivation and whether this is associated with increased adherence and engagement or whether financial incentives are necessary to promote study adherence.

#### MOST Framework

Telehealth intervention studies typically use randomized controlled trial designs. However, telehealth interventions are comprehensive packages that include components such as coaching calls, educational content, texting, email, and mobile apps. Randomized controlled trials fail to reveal which components are responsible for any behavior change elicited during the intervention; rather, they only show whether the intervention package as a whole had any effect. Therefore, it is necessary to know which elements of telehealth interventions are needed or should be removed for a more efficient telehealth intervention. To optimize the My Health, My Life, My Way program, the MOST framework will be used to assess which study elements (coaching calls and gamification) elicit behavior change. The MOST framework, derived from engineering principles, is an efficient method to gather information on each intervention component or package and investigate whether the presence or absence of a component has an impact on the performance of other components [20,21,27]. Within this study, we will assess coaching calls (scheduled or on demand) and gamification (scaled rewards or flat rewards).

### Intervention Design

The My Health, My Life, My Way study is a 2 × 2 randomized factorial experiment. By using the MOST framework, we can evaluate the effectiveness of different combinations of features within the My Health, My Life, My Way program, such as fixed versus gamified rewards and scheduled versus on-demand coaching calls. Eligible participants will be randomly assigned to one of four groups: (1) scheduled coaching calls with gamified rewards, (2) scheduled coaching calls with rewards independent of gamification, (3) no scheduled coaching calls with gamified rewards, and (4) no scheduled coaching calls with rewards independent of gamification (Table 1). For the study, 4 weeks is considered a month, thereby making the study duration 24 weeks. Study activities involving participants with disabilities will be conducted primarily on the web nationally in the United States.

**Table 1:** Research design.

Gamified rewards	Coaching calls	
	Scheduled coaching calls	No scheduled coaching calls
Gamification-based rewards	Scheduled coaching calls with gamified rewards	No scheduled coaching calls with gamified rewards
Rewards independent of gamification	Scheduled coaching calls with rewards independent of gamification	No scheduled coaching calls with rewards independent of gamification

### Sample Size

We will use a 2 × 2 factorial design for this MOST trial (detailed in the Intervention Design subsection). Our sample size and power calculations for the primary outcome (health-related quality of life) assume an overall 2-sided type I error rate of 5%, 80% power, and an analysis of covariance–based approach with 2 independent dichotomous factors (ie, each factor has 2 levels). We will assume an attrition rate of 20%, with 160 (80%) participants completing the study (referred to as completers) from 200 participants enrolled. Under these assumptions, we will have 80% power to detect at least a minimally detectable difference (Cohen *d*) of 0.36 for the main effect or 0.18 in terms of the standardized regression coefficient. In these calculations, we assume a conservative correlation of 0.6 between the baseline covariate and outcome, which is similar to that in a previous study by Froehlich-Grobe et al [4]. In addition, 12 participants will be enrolled for pilot-testing the My Health, My Life, My Way program [28].

### Recruitment

Recruitment will be conducted on the web nationally through the National Center on Health, Physical Activity and Disability (NCHPAD) website and its associated social media. The NCHPAD website has approximately 45,000 site visits per month, with most of the internet traffic flowing from the United States (74%). Through these mechanisms, interested individuals will be directed to a website landing page that will have promotional media content and information about the study. From here, participants will be directed to a separate link through the Health Insurance Portability and Accountability Act (HIPAA)–compliant REDCap (Research Electronic Data Capture; Vanderbilt University) platform [29,30] and complete a screening eligibility form. If deemed eligible, the participants will be sent an electronic consent form to be completed using REDCap. On the basis of our recruitment methods, we expect that approximately 400 individuals will be screened for eligibility.

### Allocation

Participants will be randomized into 1 of 4 study arms in a 1:1:1:1 allocation ratio using a computer-generated randomization procedure. The allocation sequence will be implemented after the baseline survey is completed. The computer-generated randomization process will be implemented in REDCap after the data from the baseline survey are completed using REDCap. This will trigger the allocation procedure to randomly assign participants to 1 of the 4 study arms. The health coach will be notified about the randomization through the telecoaching dashboard and will inform the participants about their randomization into the My Health, My Life, My Way intervention or the attention control arm during the orientation call.

### Blinding (Masking)

The principal investigator and primary statistician will be blinded to the randomization of the participants into the study arms. All other study staff will be unblinded for purposes of recruitment, consent, orientation calls, welcome calls, and coaching calls. There are no circumstances in which the principal investigator or statistician will become unblinded during this protocol.

### Eligibility Criteria

Eligible participants must meet the following inclusion criteria: (1) aged ≥18 years; (2) self-reported diagnosis of heart disease, chronic lung disease, hypertension, or type 2 diabetes; (3) living with a permanent physical disability such as spinal cord injury, spina bifida, multiple sclerosis, or stroke; (4) speak and read English; and (5) availability of a smartphone or computer. To screen for permanently impaired mobility, we will use the National Health and Nutrition Examination Survey (NHANES; physical functioning) [31].

The exclusion criteria are as follows: (1) current enrollment in any structured intervention, (2) severe cognitive impairment, (3) major cardiac event in the past 12 months, (4) resting tachycardia, (5) renal failure, (6) severe peripheral neuropathy, (7) active treatment for cancer in the past 12 months, and (8) having both visual and hearing impairments (either is acceptable).

### Participant Timelines and Intervention Duration

The study duration for those enrolled in the study will be 6 months (24 weeks), regardless of study arm allocation. Participants assigned to groups with scheduled coaching calls will receive scheduled weekly coaching calls. Those assigned to a group without scheduled coaching calls will have access to health coaches during the study and be able to communicate with the health coaches as needed.

### Study Survey Packets

After enrolling in the study, participants will automatically be electronically delivered a REDCap link to a survey packet. This survey packet will include demographics and outcome measures (Table 2). Participants will be asked to complete this survey packet 3 times during the 6-month study period (excluding the demographics in the first survey packet). The surveys should take approximately 20 minutes to complete at each time point. The time points include baseline, 3 months, and 6 months.

**Table 2:** Measures of effectiveness.

Variables^a^	Instruments	Time points
Health-related quality of life	Enabled version of 36-item Short Form Health Survey (primary outcome)	Baseline, 3 months, and 6 months
Physical activity	Godin Leisure-Time Exercise Questionnaire	Baseline, 3 months, and 6 months
Physical activity	Minutes, steps, and related data from Fitbit	Continuous
SE^b^ for managing symptoms	PROMIS^c^ Item Bank v1.0–Self-Efficacy for Managing Chronic Conditions–Managing Symptoms–Short Form 4a	Baseline, 3 months, and 6 months
SE for managing daily activities	PROMIS Item Bank v1.0–Self-Efficacy for Chronic Conditions–Managing Daily Activities–Short Form 4a	Baseline, 3 months, and 6 months
SE for managing sleep disturbance	PROMIS Item Bank v1.0–Sleep Disturbance–Short Form 4a	Baseline, 3 months, and 6 months
Psychosocial Illness	PROMIS Item Bank v1.0–Psychosocial Illness Impact-Positive–Short Form 8a	Baseline, 3 months, and 6 months
SE for managing emotions	PROMIS Item Bank v1.0–Self-Efficacy for Managing Chronic Conditions–Managing Emotions–Short Form 4a	Baseline, 3 months, and 6 months
SE for managing social interactions	PROMIS Item Bank v1.0–Self-Efficacy for Managing Chronic Conditions–Managing Social Interactions–Short Form 4a	Baseline, 3 months, and 6 months
Emotional distress	PROMIS Item Bank v1.0–Emotional Distress-Depression–Short Form 4a	Baseline, 3 months, and 6 months
SE for managing medications and treatments	PROMIS Item Bank v1.0–Self-Efficacy for Managing Chronic Conditions–Managing Medication and Treatment–Short Form 4a	Baseline, 3 months, and 6 months
SE for managing sleep-related impairments	PROMIS Short Form v1.0–Sleep-Related Impairment–Short Form 4a	Baseline, 3 months, and 6 months
Dietary intake	Short Healthy Eating Index	Baseline, 3 months, and 6 months
Medication adherence	Medication Adherence Rating Scale and Beliefs About Medication Questionnaire	Baseline, 3 months, and 6 months
Usability	Health Information Usability Evaluation Scale and System Usability Scale	Three months and 6 months

^a^In addition to the variables listed in the table, primary care visits, pharmacy visits, physical therapy and occupational therapy visits, emergency visits, and hospitalizations in the last 3 months (self-reported) will also be taken into account.

^b^SE: self-efficacy.

^c^PROMIS: Patient-Reported Outcomes Measurement Information System.

### Welcome Call

After the first survey packet is electronically delivered to participants, study team members will contact the participants by telephone. The purpose of this call is to welcome them into the program and explain the program elements. During the call, the staff will inform the participants that they will receive a technology package in the upcoming days.

### Randomization

A randomization sequence will be generated and stored within REDCap, which assigns a participant to 1 of the 4 study arms of the My Health, My Life, My Way study. Randomization will occur only after the baseline survey packet is complete.

### Overview of the Intervention

#### Orientation Call

After the welcome call and randomization, a study health coach will be notified through REDCap that a participant has been randomized to one of the study arms and will conduct an orientation call with the participant. The purpose of this call by the health coach is to introduce themselves to the participants as their primary health coach and to schedule the first weekly coaching call. For those not receiving scheduled coaching calls, the health coach will inform participants that they can contact the coaching staff during the study to ask questions related to the educational content provided on the platform.

#### Intervention Package

All participants, regardless of study arm allocation, will receive an intervention package that will include a PA tracker (Fitbit; Google LLC) and access to a telehealth platform. The Fitbit tracker will be shipped to the participants after randomization. The telehealth platform will be a central location for educational content (videos, articles, etc) for participants to access during the study. Those with conversational agents (eg, Amazon Echo) will be provided access to a My Health, My Life, My Way specific program that can operate with their home device. This package will be used for intervention delivery and not for outcome measurements.

#### Educational Content and Health Coaching for the Study Arms

The study arms for the My Health, My Life, My Way study with the coaching calls for all groups will be guided by a prepared outline of inclusive educational content that will be delivered to the participants through the telehealth platform. This content will be delivered at the beginning of each week. The educational content will be created and adapted for individuals with physical disabilities by experts and delivered to the participants as multimedia content. There will be generalized educational content applicable to all participants. On the basis of a specific physical disability as well as specific health conditions, education-specific content will be tailored to each individual. For those receiving scheduled coaching calls, the content discussed will include nutrition and eating habits, exercise and PA, and medication adherence. Questions outside the scope of the health coaching content will be dealt with by the study clinician. If the study team determines that the questions indicate that it is necessary to collect a participant’s detailed medical profile, the participant will be guided to communicate with their primary care physician. Finally, the health coach will address other health-related questions that the participant may have. For each call, the health coach will be able to record notes on the telehealth platform regarding topics discussed with the participant for future calls, as needed.

#### Telehealth Platform

The My Health, My Life, My Way telehealth platform will be used by participants throughout the duration of the study. On the platform, participants will be able to access educational multimedia content (videos and articles), track and log medication use, create and track health-related goals, and access food- and nutrition-related information. In addition, participants will be able to view upcoming health coaching calls and contact the health coaching team using the messaging feature of the platform.

### Participant Study Activities

#### Gamification Component

All participants, regardless of gamification variation, will be able to obtain points and badges on the platform. Participants can check their points status at any time during the study by logging in to the platform to view an itemized list for all badges and points. In addition, participants will be provided email notifications for all gamification updates. All activities, such as viewing and reading educational content and logging in to the platform, will automatically be updated on the platform. Health coaches will encourage participants to engage on the platform regularly. Those not receiving scheduled coaching calls who exercise the on-demand coaching option will be encouraged by a health coach to use the platform regularly.

#### Health Coaching Component

Those receiving scheduled health coaching calls will be able to view upcoming calls on the platform. In addition, email and SMS text messaging notifications will be provided to remind participants of all scheduled coaching calls. Scheduled coaching calls will occur once per week and are expected to last up to 60 minutes. Those who will not receive scheduled coaching calls will still have access to the study platform. Within the platform, participants will be able to contact health coaching staff if they desire on-demand coaching. With this on-demand coaching function, participants can discuss any questions regarding educational content on the platform.

### Exit Interviews

After the study duration, up to 30 participants from the My Health, My Life, My Way program will be contacted for a follow-up interview to obtain their experiences and feedback with the My Health, My Life, My Way telehealth platform. These interviews will be conducted on the web.

### Data Collection Methods

#### Outcome Measures

Primary and secondary outcome measures will be assessed at baseline, 3 months, and 6 months (after the intervention). The primary clinical outcome measure is health-related quality of life; the secondary outcome measures are described in Table 2 along with the primary outcome measure.

##### Health-Related Quality of Life

The primary outcome measure is health-related quality of life. This will be measured by using the 36-item Short Form Health Survey, enabled version (SF-36E). More specifically, the physical functioning section of the SF-36E has been modified, which uses disability-friendly inclusive language for individuals with mobility impairments [4].

All other measures, including demographic data and secondary outcome measures, will be retrieved and automatically stored through electronic surveys delivered by REDCap. All self-reported items within the questionnaires will be mandatory to prevent missing data. The questionnaire packet will be delivered electronically to the participants during the study.

##### PA Measurements

To measure PA, participants will complete the Godin Leisure-Time Exercise Questionnaire. This 3-item questionnaire asks questions related to strenuous, moderate, and light exercises performed during the past 7 days [32]. In addition, activity counts and time spent in PA will be collected from the participant’s Fitbit device.

##### Self-efficacy for Managing Symptoms

Participants will complete the Patient-Reported Outcomes Measurement Information System (PROMIS) Item Bank v1.0–Self-Efficacy for Managing Chronic Conditions–Managing Symptoms–Short Form 4a, which is a 4-item survey that assesses confidence in managing symptoms associated with their chronic health condition [33].

##### Self-efficacy for Managing Daily Activities

To assess confidence in handling daily activities, participants will complete the 4-item PROMIS Item Bank v1.0–Self-Efficacy for Chronic Conditions–Managing Daily Activities–Short Form 4a [33].

##### Self-efficacy for Managing Sleep Disturbances

Participants will complete the PROMIS Item Bank v1.0–Sleep Disturbance–Short Form 4a, which is a 4-item survey that evaluates sleeping habits during the previous 7 days [33].

##### Psychosocial Illness

To assess self-efficacy for managing psychosocial illness, participants will complete the PROMIS Psychosocial Illness Impact-Positive–Short Form 8a [33]. This questionnaire includes 8 items that evaluate psychosocial illness before and after an illness.

##### Self-efficacy for Managing Emotions

To assess the ability to manage emotions, participants will complete the PROMIS Item Bank v1.0–Self-Efficacy for Managing Chronic Conditions–Managing Emotions–Short Form 4a [33]. This survey includes 4 items regarding the ability to manage emotions while experiencing chronic health conditions.

##### Self-efficacy for Managing Social Interactions

Participants will complete the PROMIS Item Bank v1.0–Self-Efficacy for Managing Chronic Conditions–Managing Social Interactions–Short Form 4a [33]. This 4-item survey will assess an individual’s perceived ability to engage in meaningful social interactions.

##### Emotional Distress

To assess self-efficacy for managing depression, participants will complete the PROMIS Item Bank v1.0–Emotional Distress-Depression–Short Form 4a [33]. This survey includes 4 questions about participants’ emotions within the past week.

##### Self-efficacy for Managing Medications and Treatment

To assess confidence in managing medications and treatment, participants will complete the 4-item PROMIS Item Bank v1.0–Self-Efficacy for Managing Chronic Conditions–Managing Medications and Treatment–Short Form 4a [33].

##### Self-efficacy for Managing Sleep-Related Impairments

Participants will complete the PROMIS Short Form v1.0–Sleep-Related Impairment 4a to evaluate overall sleep quality during the previous 7-day period [33].

##### Dietary Intake

Participants will complete the Short Healthy Eating Index (sHEI) survey, which is a 23-item survey that evaluates nutritional habits, such as fruit and vegetable consumption, water intake, and sugar consumption [34].

##### Medication Adherence

To measure medication adherence, participants will complete the Medication Adherence Rating Scale (MARS). The MARS is a general, 10-item binary questionnaire that asks questions related to regularly taking medication [35]. Medication adherence will also be measured using the Beliefs About Medication Questionnaire (BMQ). The BMQ is an 18-item questionnaire that includes Likert-style questions regarding personal beliefs about how medications influence daily living [36].

##### Telehealth Dashboard Usability

To measure the usability of the telehealth dashboard, participants will complete the System Usability Scale (SUS) and the Health Information Technology Usability Evaluation Scale (Health-ITUES). These Likert scales include items asking about the dashboard’s effectiveness (ability to complete tasks), efficiency (level of dashboard use), and satisfaction (subjective reactions to the dashboard) [37,38].

##### Medication and Dosage

Participants will be asked to provide current medications and dosages throughout the study duration to control medication data during the statistical analysis.

### Feasibility Measures

This study will obtain measures related to the feasibility and acceptability of the My Health, My Life, My Way intervention on self-management for individuals with disabilities who also experience chronic conditions. Measures will include process, resource, scientific, and management feasibility outcomes. All elements of intervention delivery will be collected, including but not limited to adherence, retention, attrition, coach and participant communication needs, staff preparation, and adverse events. Obtaining these measures will inform future considerations in the delivery of a web-based self-management program for people with disabilities.

### Data Management

All data collected will be entered directly into REDCap and stored. Participants will be assigned a participant number upon enrollment in the study. Only approved study personnel will have access to the REDCap program, where data will be stored. Data checking will involve confirming that surveys, questionnaires, and health coaching calls are completed.

### Statistical Methods

All statistical analyses will be conducted in an intent-to-treat manner at the individual level taking into account the correlated nature of data (multiple time points for every participant). Statistical significance will be evaluated at a type I error rate of 5%. Quality control will include descriptive and graphical approaches to summarize baseline characteristics of all key variables. With respect to data, we will begin by systematically cleaning and verifying all collected data, as well as examining ranges, missing values, and provenance. The significance tests conducted for the primary outcome are all specified a priori and modest in number. Consistent with published guidelines for statistical reporting, exact *P* values (rather than, eg, “*P*<.05” or “NS” [not significant]) will be reported [39]. This allows readers who may have their own opinions about multiple testing corrections, configurations of a family of hypotheses, and costs of type I errors to set the significance level to know the raw, uncorrected result and make their own judgments about statistical significance [40,41]. As Saville [42] noted, in multiple comparison issues, there is no right answer, and each investigator must ultimately “cut the Gordian knot” themselves. To evaluate the potential systematic variation among the study arms, key variables such as age, race, sex, disability type, and physical function at baseline will be examined and compared for relationships with study arms. If a potential confounder is found to differ meaningfully among the groups and if we find that the inclusion of the covariate improves the corrected Akaike information criterion (AIC), it will be included as a covariate in our analyses. We will estimate and test the effects of the 4 individual intervention components by means of a full factorial experiment. First, we will fit a linear mixed model to account for correlated data, to check whether each factor has a significant effect on outcome change across the time points (baseline and 6 months). For each of the 2 components, we will determine whether there is a difference in change across time using baseline as the reference cell (ie, 6 months vs baseline). This would be considered the main effect of each component on the pre-post difference. However, statistically, these effects will be modeled as component-by-time interactions, with the 6-month outcome as the primary end point. We will also include interactions among the components (eg, factor 1 × factor 2 × time interaction). In terms of multiple testing, given that this is a pilot project (ie, an exploratory and not confirmatory trial), we will estimate the false discovery rate and false discovery proportion based on the *P* values for all outcomes.

Our 3-pronged approach involves minimizing missing data, leveraging the maximum likelihood–based approach or the multiple imputation approach under the assumptions of ignorability (ie, missing-at-random [MAR] assumption), and conducting multiple sensitivity analyses assuming MAR as well as missing not at random [43,44]. Analytically, our choice of mixed models to account for the repeated nature of the data will allow us to handle missing data for the primary and secondary outcomes under the assumptions of MAR. The MAR mechanism is ignorable when mild regularity conditions are satisfied. Such inferences can proceed by analyzing the observed data only, without explicitly addressing a form of the MAR mechanism. Missing data in other independent variables (mediators or other covariates) will be handled through the expectation-maximization algorithm coupled with bootstrapping for SEs, which will give maximum likelihood–based imputation under the MAR assumption [43].

### Data Monitoring

This project is a pilot feasibility and efficacy study that has a duration of 6 months and only involves minimal risks. Therefore, we believe that a data monitoring committee is not necessary. The principal investigator will be responsible for protocol fidelity and data collection throughout the study. The biostatistician will oversee data analysis in preparation for publication.

### Harms

The My Health, My Life, My Way study will monitor all adverse events. Adverse events will be assessed for severity and causality and reported to the institutional review board and all other relevant regulatory bodies as needed.

### Auditing

All elements of the study protocol will be evaluated at regular intervals (ie, weekly, monthly, or quarterly) to ensure proper and consistent adherence to the study design and implementation. Elements of auditing will include checklists; coaching call logs; audio recordings or coaching calls; content resource banks; telehealth platform reviews and event logs; review of participant food, PA, and medication, as well as time spent on the platform and in regular team meetings to discuss participant progress; educational content provided during health coaching calls; and protocol adherence.

### Protocol Amendments

Any protocol changes deemed necessary by the principal investigator or study team will be submitted as amendments to the university’s institutional review board, and we will only apply changes after approval.

### Ethics Approval

This protocol has been approved by the institutional review board of the University of Alabama at Birmingham (IRB-300009485).

### Consent or Assent

All consent processes will be conducted on the web through predetermined screening questionnaires provided through the website landing page and REDCap link. If a participant is deemed eligible through the web-based process, an electronic consent form will be sent to the participant to complete. Only approved study staff will have access to completed records of the consent form.

### Confidentiality

Personal information will be collected in this study. All personal information shared, such as demographic details, will be collected and stored through REDCap. Only approved study staff will have access to securely stored personal information. All information will be subject to the university’s institutional review board and the policies of affiliated entities surrounding confidentiality.

## Results

### Overview

The My Health, My Life, My Way protocol was funded in September 2020 and has been approved by the university’s institutional review board and registered at ClinicalTrials.gov (NCT05481593). The telehealth platform (dashboard and educational content) and website landing page are being developed by the study technical support team. Research personnel are developing the database (REDCap) that will store data and communication mechanisms (ie, emails and SMS text messaging notifications). We project that enrollment for the study will begin in May 2023, with data analysis being completed within 6 months of completing data collection.

### Dissemination Policy

The results of this study will be disseminated publicly through publication in peer-reviewed journals and presented at regional and national conferences.

## Discussion

### Many Important Implications

The aim of this paper was to detail the study protocol used to develop a web-based self-management program for individuals with physical disabilities who also experience chronic conditions by using artificial intelligence techniques. The incorporation of variations in coaching calls and gamification will be evaluated to determine which combination of intervention components is the most effective for personal self-management. As the My Health, My Life, My Way program will be implementing a 4-arm protocol using the MOST design, many important implications can be drawn based on the findings of this study. The findings will assess what resources are required by self-management programs to maintain high levels of adherence and engagement. For coaching calls, if scheduled coaching calls are not as effective at promoting self-management as on-demand coaching calls, this would result in a reduced demand for excess resources, including the number of health coaches, monetary expenses, training, and daily availability of health coaches. If scheduled coaching calls are shown to be more effective than on-demand coaching calls, future web-based self-management programs should consider bolstering the resources to provide appropriate health coaching with qualified health coaches. Within the gamification context, the results from this study will inform future programming for individuals with physical disabilities who also experience chronic conditions. If gamified rewards are shown to be more effective than the fixed rewards model, self-management programs should consider using appropriate methods to promote extrinsic motivation by providing desirable incentives for behavior change. If fixed rewards are shown to be more effective than gamified rewards, this could indicate that a participant’s intrinsic motivation to improve their self-management is more important than extrinsic rewards such as redeeming increasing monetary incentives to promote behavior change.

As multiple barriers hinder a person’s ability to successfully manage chronic conditions, My Health, My Life, My Way aims to create a sustainable, scalable, accessible, implementable, sustainable, and inclusive self-management program for people with disabilities, which currently does not exist. This study will examine the feasibility and preliminary efficacy of My Health, My Life, My Way for individuals with physical disabilities who also experience chronic conditions using mobile apps and web-based applications, telehealth coaching sessions, and educational multimedia material. As part of the development process of My Health, My Life, My Way, we have conducted extensive focus groups with stakeholders with physical disabilities who also experience chronic conditions to obtain their perspectives on the proposed program and plan to implement relevant changes during further development. In addition, in line with the protocol, My Health, My Life, My Way is built upon a prior protocol evaluating the effectiveness of a web-based diabetes management program for individuals with physical disabilities [45]. The My Health, My Life, My Way study will also focus on quality-of-life measures during the intervention and will include qualitative interviews conducted after the intervention to provide a holistic evaluation of the program as a viable mode for self-management of chronic conditions. Next, to reduce barriers experienced by people with disabilities, the My Health, My Life, My Way program will be conducted completely on the web, with all coaching sessions taking place over the telephone, thereby allowing all participants to engage in the program and manage their health conditions in a home environment.

### Limitations and Strengths

This protocol includes limitations. First, we are using self-reported measures to collect data on participant perceptions about their physical and mental health. Therefore, we are relying on participants’ responses to be truthful when they answer all the surveys. The surveys we will use in this study have been shown to be reliable and valid in assessing physical and mental health outcomes. Second, My Health, My Life, My Way is using a web-based platform that participants will use to access multimedia content; therefore, those without internet capabilities will be unable to participate.

This study will add to the current literature to improve the quality of web-based self-management programs. In addition, not only will My Health, My Life, My Way determine how coaching calls and gamification variations affect behavior, but this program is also designed to be inclusive for those who experience physical disabilities, considered an underserved population. Finally, the findings from this study will inform how future self-management programs can be created and tailored to populations consisting of individuals with disabilities.

## Appendix

Multimedia Appendix 1 Peer-review report by the National Institute on Disability, Independent Living, and Rehabilitation Research (NIDILRR) - Disability and Rehabilitation Research Program (DRRP) (Washington, DC).

## Abbreviations

AIC: Akaike information criterion
BMQ: Beliefs About Medication Questionnaire
CDC: Centers for Disease Control and Prevention
Health-ITUES: Health Information Technology Usability Evaluation Scale
HIPAA: Health Insurance Portability and Accountability Act
MAR: missing at random
MARS: Medication Adherence Rating Scale
MOST: multiphase optimization strategy
NCHPAD: National Center on Health, Physical Activity and Disability
NHANES: National Health and Nutrition Examination Survey
PA: physical activity
PROMIS: Patient-Reported Outcomes Measurement Information System
REDCap: Research Electronic Data Capture
SF-36E: 36-item Short Form Health Survey, enabled version
sHEI: Short Healthy Eating Index
SPIRIT: Standard Protocol Items: Recommendations for Interventional Trials
SUS: System Usability Scale

## Data Availability

The data generated or analyzed during the study will be available from the corresponding author on reasonable request.
